# Efficient extraction of aromatic amines in the air by the needle trap device packed with the zirconium based metal–organic framework sorbent

**DOI:** 10.1039/d0ra00687d

**Published:** 2020-04-03

**Authors:** Ali Akbar Alinaghi Langari, Ali Firoozichahak, Saber Alizadeh, Davood Nematollahi, Maryam Farhadian

**Affiliations:** Student Research Committee, School of Public Health, Bam University of Medical Sciences Bam Iran; Department of Occupational Health, Faculty of Health, Social Determinants of Health Research Center, Gonabad University of Medical Science Gonabad Iran a.firoozi@edu.umsha.ac.ir; Department of Chemistry, Bu-Ali-Sina University Hamedan Iran; Department of Biostatistics, School of Public Health and Research Center for Health Sciences, Hamadan University of Medical Sciences Hamadan Iran

## Abstract

In this study, development of a needle trap device (NTD) packed with UiO-66 adsorbent was used for the sampling of the aromatic amine compounds (including aniline, *N*,*N*-dimethylaniline and *o*-toluidine) followed by gas chromatography (GC) with flame-ionization detector (FID) analysis. The UiO-66 sorbent was synthesized and then packed inside a spinal needle (Gauge 22). The synthesized sorbent was characterized with the XRD, FE-SEM, EDS and FT-IR techniques. This study was conducted both in the laboratory and in the real samples. In the laboratory, the sampling parameters (such as temperature and humidity) and desorption parameters (including desorption temperature and desorption time) were optimized using Response Surface Methodology (RSM) by Central Composite Design (CCD). The results indicated that the performance of the sampling device decreased with increasing the sampling humidity and temperature. Moreover, the highest peak area responses of the studied analytes were observed at a desorption time of 3 minutes and desorption temperature of 270 °C. The values of the limit of detection (LOD) and limit of quantitation (LOQ) were in the range 0.01–0.02 and 0.03–0.05 ng mL^−1^, respectively. Our findings demonstrated that NTD packed with synthesized UiO-66 has good repeatability (RSD = 1.3–6.8%) and acceptable reproducibility (with three NTDs) (RSD = 1.3–9.7%). Comparison of the results between NTD-UiO-66 and NIOSH2002 showed a sufficient correlation (0.98–0.99) between two methods. Therefore, the results indicated that the NTD packed with the UiO-66 adsorbent can be used as a powerful technique for occupational and environmental monitoring.

## Abstract 2

Development of a needle trap device (NTD) packed with UiO-66 adsorbent was used for the sampling of the aromatic amine compounds (including aniline, *N*,*N*-dimethylaniline and *o*-toluidine) followed by gas chromatography (GC) with flame-ionization detector (FID) analysis.

## Graphical abstract

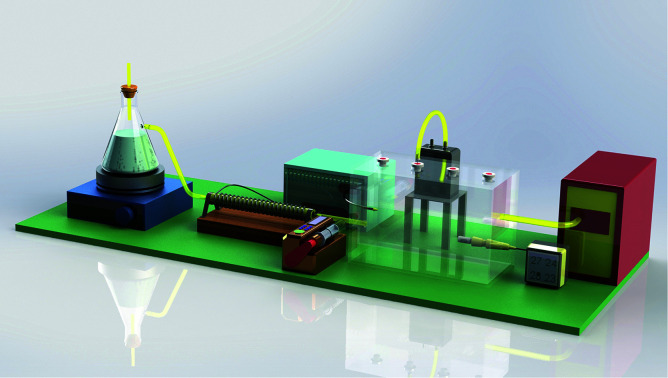

## Introduction

Aromatic amine compounds are a large group of compounds that are widely used in industrial and non-industrial applications. These compounds are used in pesticides, rubber, pharmaceuticals and leather factories. Aromatic amines are also found in wood-burning, tobacco smoke and diesel exhaust. The widespread use of these compounds increases their occupational and environmental exposure.^1^ Previous studies have investigated the effects of aromatic amines on human health and the environment, and it has been reported that the long-term exposure to these compounds is associated with a wide range of health problems, such as bladder and breast cancers, negative effects on fertility and behavioral problems.^2–4^

The Occupational Safety and Health Administration (OSHA) and the American Conference of Governmental Industrial Hygienists (ACGIH) has established the permissible limit of 2.0 ppm for Aniline, and 5.0 ppm for *N*,*N*-dimethylaniline. Therefore, developing a method with high sensitivity and precision for determining trace levels of these pollutants in the air is very important.

Various methods have been recommended by NIOSH (2002) and OSHA (65) for monitoring aromatic amine compounds in the air, which most of them are based on the use of a surface sorbent, extraction and preparation with an organic solvent. In these solvent-based methods, the analyte recovery is not complete and the use of organic solvents is associated with so many problems. Most of the organic solvents are toxic and carcinogen and can lead to several environmental problems that are considered as the main disadvantages of these techniques.^5^ Moreover, the adsorbents used in these methods have disadvantages such as low sensitivity and lack of selectivity.

The NTD was first introduced by Pawliszyn in 2001,^6^ and due to its advantages for the sampling of pollutants in the air, have attracted much attention from researchers in recent years.

In these methods have overcome the need for using organic solvents. The pre-concentration, preparation and analysis steps are conducted in a single step. Furthermore, these methods provide high accuracy and precision in addition to reducing time and costs.^7–9^

The type of absorbent used in the NTD plays a key role in its performance for the sampling and analysis of compounds of interest. Many studies have investigated the effect of commercial and synthetic adsorbents in NTD for detecting various chemicals.^10–12^

The MOFs are new porous structures that its principal was introduced in the early 1990s by Rabson and Hoskins.^13^ These frameworks have an organic–mineral hybrid nature that has the properties of both organic and inorganic elements.^14–16^ Because of the presence of the same units in their structures, they have regular and uniform cavities.^17^ This is an important factor in increasing selectivity and distinguishing these materials from carbon and zeolite materials.^18,19^ Recently, there has been increasing interest in applying MOFs with porous structure and high adsorption capacity as adsorbents in solid-phase extraction techniques.^20–22^ Zr_6_O_4_(OH)_4_(BDC)_6_ framework, marked UiO-66 is one of the high-valent metal ion MOFs that offers superior chemical and thermal stabilities.^23^ The various applications as so as catalysis, photocatalysis, supercapacitors, micro-extractions have been reported based on the interesting properties of UiO-66.^24–27^ From the environmental standpoint, one of the important potential applications of this MOF is adsorption of the VOCs because of high surface area and suitable adsorption and recovery.^28–31^

Until now, there is no study using UIO-66 sorbents for aromatic amines in NTD for monitoring compounds in the air. Therefore, in this paper, NTD packed with UiO-66 adsorbent was used for the sampling of aromatic amines (including Aniline, *N*,*N*-dimethylaniline and *o*-toluidine) followed by GC analysis. The effects of some parameters, such as sampling temperature, relative humidity, desorption temperature and time were investigated on the performance of the proposed NTD. In order to estimate the optimal values of the mentioned parameters, the Response Surface Methodology (RSM) and Central Composite Design (CCD) were applied. Moreover, in order to validate the NTD performance some analytical parameters, such as carryover effect, Limit of Detection (LOD), Limit of Quantitation (LOQ), and Linear Dynamic Range (LDR) were investigated.

## Experimental

### Chemicals and reagents

Aromatic amines including aniline (99%), *N*,*N*-dimethylaniline (98%) and *o*-toluidine (99%) with high purity and ethanol were obtained from Merck (Darmstadt, Germany). In order to synthesize UiO-66 adsorbent, *N*,*N*-dimethylformamide (99%), terephthalic acid (98%), acetone and ZrCl_4_ (98%) were purchased from Sigma Aldrich. And solid sorbent tube (silica gel, 150 mg/75 mg) were obtained from SKC.

### Instruments

A 22-gauge spinal needle (9.0 cm in length and 0.71 mm OD) was purchased from Kosan Ltd. (Japan-Tokyo). A digital hygrometer (Testoterm model, GmbH Company) was used to monitor the percentage of relative humidity inside the standard chamber. A low-flow personal sampling pump (SKC 224-3) was applied to sample the analytes from the standard chamber. Moreover, a high-flow vacuum pump (Flite 3 high-volume sample pump, SKC) was used to provide a constant flow. A 5.0 mL medical syringe was also applied for delivering analytes, and a 50.0 mL medical syringe was used in a pump syringe to inject the analytes into the standard chamber (Mina Tajhiz Company, Tehran, Iran). The syringe pump (Sp 1 Plus model, Hiroshima, Japan) was used to inject the analytes at a specified rate into the standard chamber. For a sampling of the analytes under predetermined conditions, a home-made glass chamber equipped with temperature and humidity sensors were used.

### Gas chromatography analysis

Varian CP-3800 gas chromatograph with flame ionization detector (FID) equipped with a CP-Sil 8 CB capillary column was used for the analysis of aromatic amines. The GC device was operated under splitless mode. Nitrogen gas with a purity of 99.99% was used as a carrier gas. The temperature of the column started at 50 °C and kept for 1 min then increased to 220 °C with a rate of 10 °C min^−1^ and held steady for 10 minutes. The total temperature program was 28 min. The detector temperature was set at 250 °C and the injection port temperature was adjusted between 220–280 °C. X-ray diffraction patterns were recorded using a Rigaku Ultima IV device (Japan). In order to investigate the stability of the crystal structure, transmission mode with Cu Kα radiation at 2*θ* values = 5–80 was used. Absorbent morphology was examined using a scanning electron microscope (SEM, TESCAN mira3, Czech Republic). Moreover, an Elmer Spectrum 65 Fourier Transform Infrared (FT-IR) spectrometer (PerkinElmer, USA) was applied to verify the bonds in the adsorbent structure.

### Synthesis of UiO-66

The UiO-66 adsorbent was synthesized according to the method introduced by Michael J. Katz *et al.*^32^ Briefly, 250.0 mg of zirconium chloride, 2.0 mL of HCl and 10.0 mL of DMF was poured into 50 mL flask-glass and sonicated for 20 min. Then, 246.0 mg of terephthalic acid and 20.0 mL of DMF were added to the solution. The suspension was again sonicated in the ultrasonic bath for 20 min. Then, the flask containing the mixture was placed in an oven at 80 °C for 12 hours. In the next step, the flask was placed in the oven at 120 °C for six hours. Reactions were performed in hydrothermal conditions. Afterwards, a two-phase solution was obtained, which the upper phase was removed, and then 80 mL of ethanol was added to the lower phase and placed in an oven at 60 °C for 48 hours for drying. Finally, the obtained solution was poured into a crucible, and placed in an oven at 150 °C for two hours. Eventually, the UiO-66 adsorbent has appeared as white crystalline powders.

### Pilot study

**Fig. 1:**
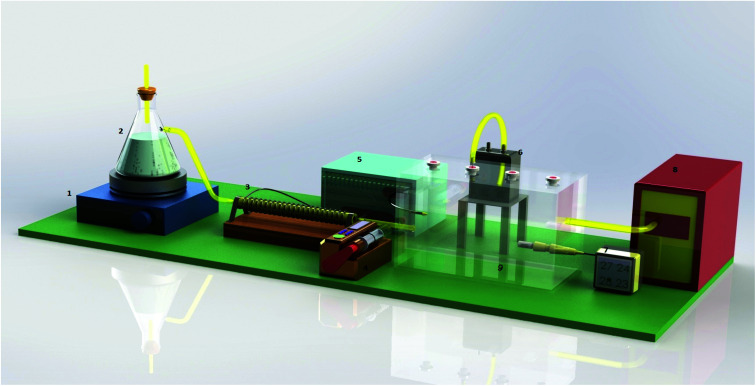
Schematic shape of the sampling chamber. (1) Heater. (2) Glass flask to produce steam. (3) The thermal winding for heating inside the chamber. (4) A syringe pump (JMS SP-510, Hiroshima, Japan) to insert the analytes into the standard chamber. (5) Thermostat to adjust the temperature inside the standard chamber. (6) Low-flow sampling pump (SKC 222–3). (7) Hygrometer (Model Testoterm GmbH Co.). (8) High-flow vacuum pump (BioLite Sampling pump, SKC), and (9) glass chamber made to provide constant sampling conditions.

The schematic of the pilot study is shown in Fig. 1. As can be seen from this figure, the pilot study consisted of different parts including environmental vacuum pump, standard glass chamber, digital pump syringe, thermostat and dimmer, thermal winding, magnetic and hot plate. The parameters studied in this research, which their effects were investigated on the performance of the proposed NTD during the sampling phase, included analyte type, sampling temperature, and relative humidity. In the present study, three types of aromatic amines were studied. The desired concentrations of the studied compounds were obtained inside the dynamic standard chamber using a pump syringe. The concentrations of the analytes were determined and calibrated in accordance with the NIOSH method (NIOSH 2002). The analyte concentration inside the standard chamber was set at 1.0 ng mL^−1^.

In the present study, the sampling performance of the proposed NTD was evaluated under three sampling temperatures (15, 30 and 45 °C), which represent cold, moderate, and warm temperatures in the workplace. A temperature sensor and thermal winding were used to adjust the sampling temperature inside the standard chamber. Adjusting the relative humidity inside the standard chamber was conducted manually. For this reason, a steam generator and digital hygrometer were used. The sampling of the target analytes was conducted under three levels of relative humidity including 20, 45 and 70%, which indicate low, moderate and high humidity, respectively. The sampling flow rate through the packed NTD was 3.0 ± 0.5 mL min^−1^.

### NTD preparation

**Fig. 2:**
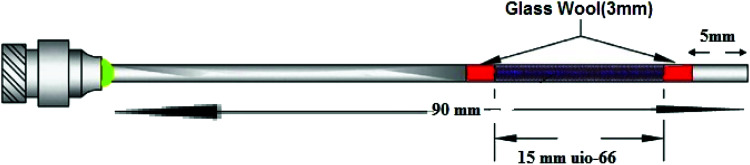
NTD designed for sampling of aromatic amines.

A gauge-22 spinal needle was used as the main body of NTD. The UiO-66 adsorbent was packed into the spinal needle. For this purpose, a length of 1.5 cm of the needle was packed with the synthesized sorbent and both sides of the packed sorbent were fixed by placing a double layer of glass wool (in length of three mm). In this method, the adsorbent is packed inside the needle with two layers of glass wool before and after the adsorbent. After the second layer of the glass wool, five mm from the tip of the needle was empty. The schematic of NTD is shown in Fig. 2. This free space was embedded to prevent falling the sorbent particles inside the GC injector during injecting NTD into the GC instrument. In order to prevent clogging inside the NTD, the sorbent was mixed with various ratios of glass powders. In this step, the effects of various ratios of the sorbent to glass powder (1 : 1, 2 : 1, 3 : 1) were tested on the NTD performance. The highest peak area responses of the analytes with the proposed NTD were observed in the ratio of 1 : 1 of (sorbent: glass powder). It is noteworthy, diagnostic tests proved the glass powders didn't have any effected on the extraction capacity of sampling and analysis.

The amount of adsorbent packed inside the NTD was about 1.0 mg. A total of 2.0 mg of absorbent mixed with glass powder was packed inside the NTD. After packing, the passing flow rate through the packed NTD was determined and calibrated while it was connected to a low-flow personal sampling pump (SKC 222 series, PA, USA).

Three mL of pure nitrogen gas was drawn using a five mL medical syringe from a Tedlar bag that was previously filled with nitrogen gas. The packed NTD connected to a medical syringe was placed at the injection port (1–4 min). After desorption time, the desorbed analytes were delivered into the GC column by injecting the carrier gas (N_2_) with a constant flow inside the medical syringe.

### Experiments

In this study, the number of tests and the optimal values of different variables was determined using response surface methodology (RSM) by central composite design (CCD). RSM consists of statistical and mathematical methods that are used in experimental research to estimate the relationship between one or more response variables and several independent variables through a set of designed experiments and regression analysis methods.^33^ The laboratory data were analyzed by design expert software (version 10).

### Desorption temperature and time

The temperature of the GC injection port determines the desorption temperature. By placing the adsorbent containing the target analytes into the injector port, the desorption has occurred, and then the trapped analytes are separated from the adsorbent bed and directed into the GC column. Determining the optimal desorption temperature is very important because each absorbent requires a specific desorption temperature, and the analyte desorption is not completely carried out at lower temperatures resulting in carryover effect. On the other hand, increasing desorption temperature over the optimal temperature in the injection port may destroy the absorbent structure and reduce its life span. In this study, the effect of desorption temperature was investigated at three levels of 200, 240 and 280 °C. Desorption time is the optimal time required for the complete desorption of compounds of interest from the adsorbent bed. At lower than this time, desorption is incomplete and higher times are not necessary. The high temperatures may cause damage to the absorbent and decreases its life span. In this study, the effect of desorption time was tested on the NTD performance at three levels of 2, 3 and 4 minutes.

### Carryover measurement and memory effect

The memory effect is the amount of analyte that remains on the sorbent bed after adsorption and separation of the compounds of interest. Reusing the same adsorbent for the sampling of the desired compounds of interest causes an error in the results. In order to determine the amount of memory effect, the sampling was carried out under the optimal conditions, and then the NTD was injected into the GC system based on the optimal desorption temperature.

### Storage time

The storage duration period of a sampler is one of the functional parameters, which is equal to the maximum storage time that a certain concentration of the trapped analyte can remain stable on the adsorbent surface without mass loss. In this study, to evaluate the storage time, a specific concentration of the analytes (1.0 ng mL^−1^) was first sampled from the standard chamber. Then, both sides of the NTD were sealed with parafilm. The sealed NTD was placed in a glass container. The storage stability of the NTD: UIO-66 was checked out in the range 1–30 days at room temperature (25 °C) and refrigerator (4 °C) temperatures. Afterwards, the stored samples were analyzed to determine the storage capability of the NTD.

### Method validation

The validation of the NTD technique was performed under optimized conditions for quantitative analysis of target compounds in a standard chamber. The calibration curve was drawn for a concentration range of 0.03 to 200.0 ng mL^−1^. The linearity of the calibration curve was determined with regard to correlation coefficients. The specified concentrations were injected into the chamber using a pump syringe. The concentration of the analytes of interest inside the sampling standard chamber was determined with the NIOSH method 2002. The LOD, LOQ and LDR method was also evaluated for validation of proposed. To determine LOD and LOQ values, the concentrations of the analytes inside the standard chamber were gradually decreased by dilution to reach concentrations corresponding the signal to noise ratios of 3 and 10, respectively. The (LDR) was also obtained by increasing the concentration of the analytes with a linear relationship between enhancing the concentration and the peak area. In evaluating the analytical functions, a technique with lower LOD and LOQ and broader LDR offer better performance.

### Repeatability and reproducibility

In this study, to obtain the repeatability and reproducibility of the proposed technique, relative standard deviation (RSD) was used. The repeatability was calculated as RSD percentage for the results of the sampling from different concentrations with an NTD, and the reproducibility was also obtained by calculating the RSD percentage for the results of the sampling from a given concentration with three NTDs.

### Real sampling analysis

After optimization of the laboratory circumstance and determination of effective parameters on the NTD packed UiO-66 performance, real sampling of the plastic industry containing aniline and *N*,*N*-dimethylaniline was performed by the NTD packed UiO-66. Finally, the collected data through the obtained results of the NTD packed UiO-66 absorbent compared with Standard method (NIOSH-2002).

## Results and discussion

### UiO-66 adsorbent properties

**Fig. 3:**
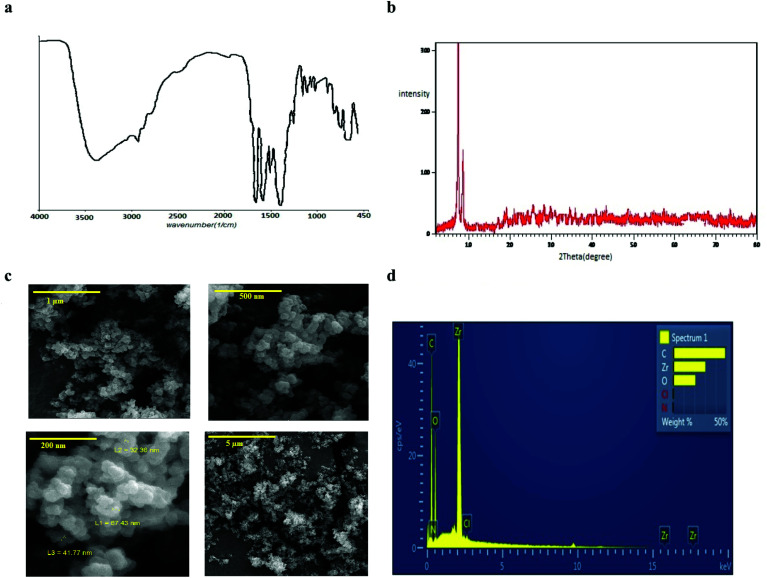
Characteristics of UiO-66: (a) IR spectrum, (b) XRD pattern, (c) FE-SEM imagine, (d) EDS patterns.

In order to evaluation of the functionality and bonding groups of prepared UiO-66, FT-IR analysis was used (Fig. 3a). Based on the reported documents^34,35^ the presence of characteristic coupled peaks of asymmetric and symmetric vibration carboxylate at the 1622–1578 and 1443–1374 cm^−1^ proved the coordination of terephthalate anion with the zirconium cation.

Also, the *ex situ* powder X-ray diffraction pattern (Fig. 3b) illustrated well-defined crystallinity of the synthesized UiO-66 with the characteristic diffraction peaks in the 2*θ* 5.7° and 9° which is consistent with the results of previous studies.^36,37^

Also, Fig. 3c and d show the recorded FE-SEM image of the UiO-66 crystals and EDS analysis of them that proved the accuracy of synthesized MOF. The weight percentage of the elements were attributed to carbon, zirconium and oxygen, respectively. Also, the specific surface area of UIO-66 was 1047 m^2^ g^−1^, indicating the high surface area and porosity of the adsorbent.^38^

### Investigation of the effect of environmental conditions on sampling efficiency

To determine the effect of environmental parameters on sampler performance, the influence of sampling temperature was studied at three levels (15, 30 and 45 °C) and the influence of relative humidity was investigated at three levels (20, 45 and 70%). Eventually, the optimal sampling conditions were determined for the proposed NTD. The levels of experiments are presented in Table 1. The results of the analysis with the RSM and CCD showed that the quadratic model was meaningful for these data and has higher *R*^2^ value than the linear and 2FI models. The modelling results of the sampling parameters are shown in Table 2.

**Table 1:** Number of experiments and different levels of sampling and desorption parameters

Variable			Unit	Level number	Variable levels		
Sampling variables	Sampling temperature	A: temperature	°C	3	15	30	45
	Relative humidity	B: humidity	%	3	20	45	70
Desorption variables	Desorption time	C: time	min	3	2	3	4
	Desorption temperature	D: temperature	°C	3	200	240	280

**Table 2:** Optimal values and ANOVA results of sampling and desorption variables

Source	Sum of squares	d*f*	*f*-Value	*p*-Value	Source	Sum of squares	d*f*	*f*-Value	*p*-Value
Sampling variables					Desorption variables				
Aniline									
Model	1.261 × 10^7^	5	30.88	0.0001	Model	1.361 × 10^8^	5	40.12	<0.0001
A	1.087 × 10^6^	1	13.32	0.0082	C	1.762 × 10^8^	1	32.42	0.0002
B	7.880 × 10^6^	1	96.52	< 0.0001	D	2.599 × 10^8^	1	47.82	<0.0001
AB	1.573 × 10^6^	1	19.26	0.0032	CD	4761.00	1	132.23	<0.0001
A^2^	1.805 × 10^5^	1	20.21	0.0807	C^2^	5.031 × 10^5^	1	832.25	<0.0001
B^2^	2.049 × 10^6^	1	25.10	0.0015	D^2^	3.325 × 10^6^	1	10.23	0.0121
*R* ^2^ = 0.95 optimal relative humidity (%) = 20					*R* ^2^ = 0.88 optimal time (min) = 3				
Adj *R*^2^ = 0.92 optimal temperature (°C) = 15					Adj *R*^2^ = 0.86 optimal temperature (°C) = 270				

*N*,*N*-Dimethylaniline									
Model	1.398 × 10^8^	5	29.16	0.0002	Model	4.519 × 10^8^	2	108.52	<0.0001
A	9.513 × 10^6^	1	9.92	0.0161	C	1.676 × 10^8^	1	80.48	<0.0001
B	9.006 × 10^7^	1	93.94	<0.0001	D	2.843 × 10^8^	1	136.55	<0.0001
AB	1.463 × 10^7^	1	15.27	0.0058	CD	3.224 × 10^5^	1	141.2	<0.0001
A^2^	8.335 × 10^6^	1	8.70	0.0214	C^2^	4.056 × 10^7^	1	192.88	<0.0001
B^2^	2.438 × 10^7^	1	25.43	0.0015	D^2^	2.342 × 10^7^	1	89.32	<0.0001
*R* ^2^ = 0.95					*R* ^2^ = 0.95 optimal time (min) = 3				
Adj *R*^2^ = 0.92 optimal relative humidity (%) = 20					Adj *R*^2^ = 0.94 optimal temperature (°C) = 270				
Adj *R*^2^ = 0.96 optimal temperature (°C) = 16.29					Adj *R*^2^ = 0.74				

*o*-Toluidine									
Model	1.195 × 10^9^	5	143.97	<0.0001	Model	1.375 × 10^9^	5	750.77	<0.0001
A	2.303 × 10^8^	1	138.68	<0.0001	C	2.870 × 10^8^	1	783.67	<0.0001
B	6.377 × 10^8^	1	384.02	<0.0001	D	9.522 × 10^8^	1	2600.05	<0.0001
AB	2.151 × 10^8^	1	129.54	<0.0001	CD	2.884 × 10^5^	1	231.2	<0.0001
A^2^	2.950 × 10^7^	1	17.76	0.0040	C^2^	6.078 × 10^7^	1	165.98	<0.0001
B^2^	1.099 × 10^8^	1	66.17	<0.0001	D^2^	2.509 × 10^7^	1	68.52	<0.0001
*R* ^2^ = 0.99 optimal relative humidity (%) = 20					*R* ^2^ = 0.99 optimal time (min) = 2.98				
Adj *R*^2^ = 0.98 optimal temperature (°C) = 15					Adj *R*^2^ = 0.98 optimal temperature (°C) = 269.36				

The results indicated that the effects of sampling variables (sampling temperature: A and relative humidity: B) and their second-order effects including A^2^ and B^2^ were significant. The same trend was also observed for the desorption variables (desorption time: C and temperature: D).

**Fig. 4:**
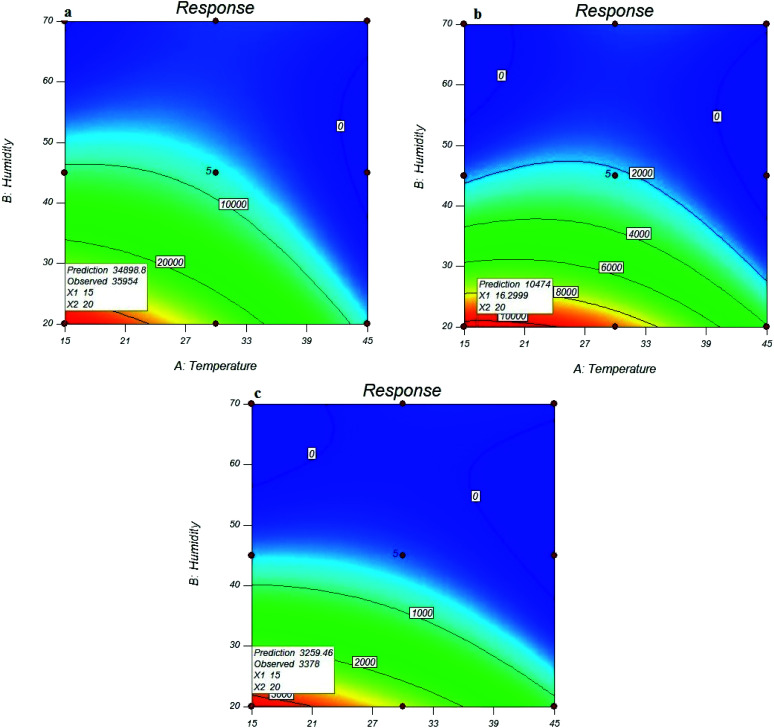
Effect of sampling conditions on the efficiency of NTD packed with UiO-66 in the extraction of *o*-toluidine (a), *N*,*N*-dimethylaniline (b), and aniline (c).

As shown in Fig. 4, the maximum peak area response of the analytes of interest was obtained at the lowest sampling temperatures (15 °C). The sampling efficiency was reduced with increasing temperature, which indicated the interference effect of temperature on the trapping and adsorbing function of the MOF sorbent. This can be due to the vapour pressure of each compound that is very close to the sampling temperature, and the vapour pressure also increases with increasing temperature for each analyte. Increasing the vapour pressure of the analyte and increasing the molecular movement can cause a reduction in the efficiency of the performance of the surface adsorbents.

Similar results were also obtained for the relative humidity. As shown in Fig. 3, the peak area response of the target analytes, which indicated the functional efficiency of the sampler, was decreased by increasing the relative humidity. The reason is attributed to the role of moisture content of the air passing through the needle that can cause clogging inside the NTD. Fig. 4 shows the predicted *versus* observed values and optimal values.

### Check the time and temperature desorption

**Fig. 5:**
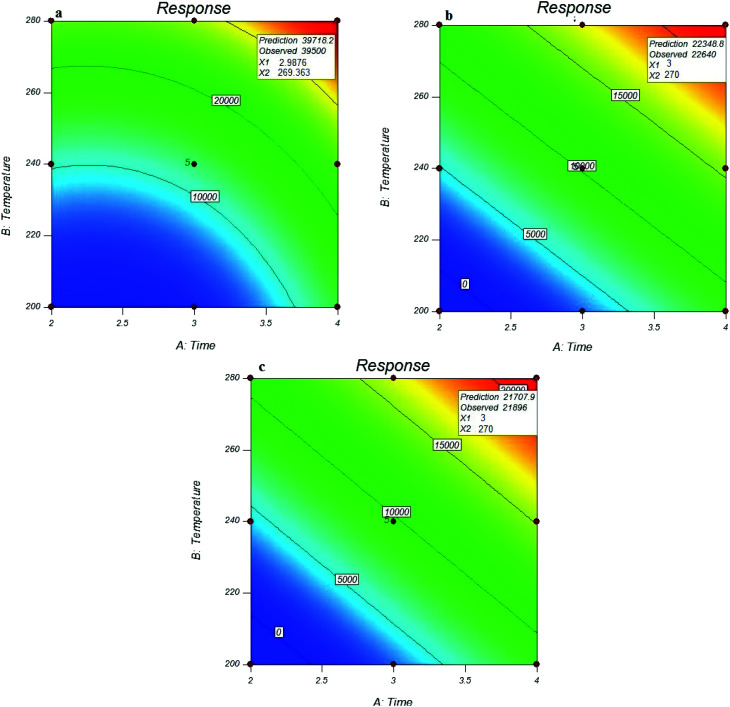
Effect of desorption parameters on the efficiency of NTD: UiO-66 in the extraction of *o*-toluidine (a) *N*,*N*-dimethylaniline (b) and aniline (c).

For this purpose, the influence of desorption time at three levels of 2, 3 and 4 minutes, and desorption temperature at three levels of 220, 240 and 280 °C were investigated on the NTD performance. The number of experiments with different desorption times and temperatures is presented in Table 1. The optimal values of desorption time and temperature were estimated using the linear model by RSM. The results showed that the optimal desorption time for all analytes was 3 minutes and the optimal desorption temperature was 270 °C. The results showed that the desorption rate increases with increasing the desorption temperature. It should be noted that the peak area responses at 280 °C were the same as 270 °C. To allow less stress to the adsorbent, 270 °C was considered as the optimum temperature. The optimal values of desorption time and temperature for all analytes are presented in Fig. 5. These results are consistent with previous studies. For example in the study of Poormohammadi *et al.*, amberlite XAD-2 resin was used as a sorbent in NTD for the extraction of aromatic amines in air, the desorption temperature was estimated to 280 °C.^3^ Or, in Huang Minjia, study, polyaniline coating was used in SPME to determine the aromatic amines with a desorption temperature of 280 °C.^39^ The modelling results showed that the coefficients of desorption temperature and time were significant for all studied analytes. In addition, there were significant coefficients for the squares of the two variables (desorption temperature and time). The modelling results of desorption parameters are shown in Table 2. The difference in the desorption temperature and time between different analytes can be attributed to the various boiling points of these compounds.

**Fig. 6:**
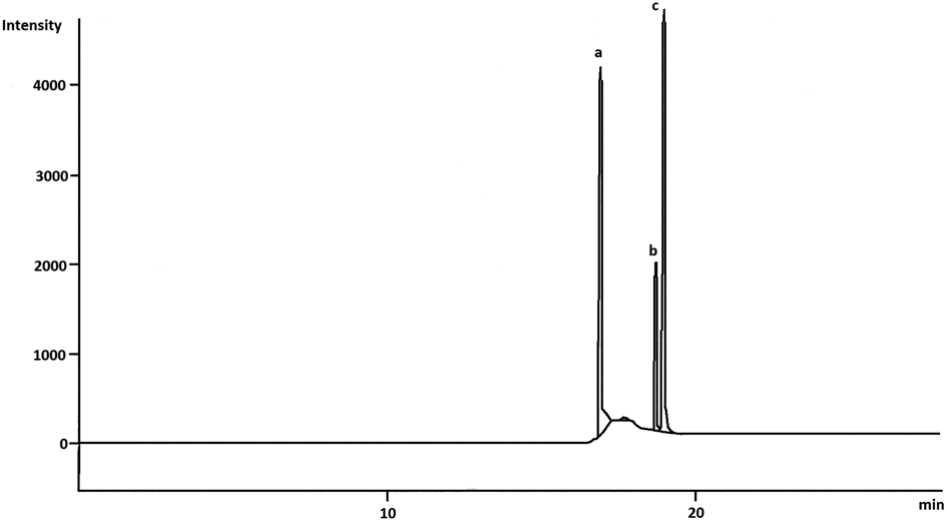
Chromatogram obtained with needle trap sampling of the aniline (a), *o*-toluidine (b) and *N*,*N*-dimethylaniline (c) concentration: ng mL^−1^.

The chromatograms obtained from the needle trap sampling were shown in Fig. 6 from the pilot study in the lab.

### Carryover effect

The carryover effect has a key role in the required time for the conditioning and preparation of the NTD. It also interferences with the further applications of NTD as a reusable method. In general, the carryover effect depends on desorption time and temperature. In this study, the carryover effect was not observed at the optimal levels (desorption time: 3 min and temperature: 270 °C). Our findings are consistent with the similar studies. For example In Sarafraz-Yazdi, *et al.*, study, the PEG and PEG/CNTs sol–gel fiber was used as a coating SPM fiber for the determination of aromatic amines with desorption temperature of 280 °C, no carryover effect was observed.^40^ Moreover, in a similar study, hydroxydibenzo-14-crown-4(OH-DB14C4) coating was used in SPME to analyze of aromatic amines, no memory effect was observed.^41^ The carryover effect was also investigated at the other desorption times (1, 2 and 3). The results are presented in Table 3.

**Table 3:** The effect of carryover effect at different desorption times

Desorption time (min)	Carryover effect for each analyte (%)		
	Aniline	*o*-Toluidine	*N*,*N*-Dimethylaniline
1	0.54	0.65	0.31
2	0.36	0.39	0.19
3	0.21	0.28	0.1
4	NA	NA	NA

### Storage time

**Fig. 7:**
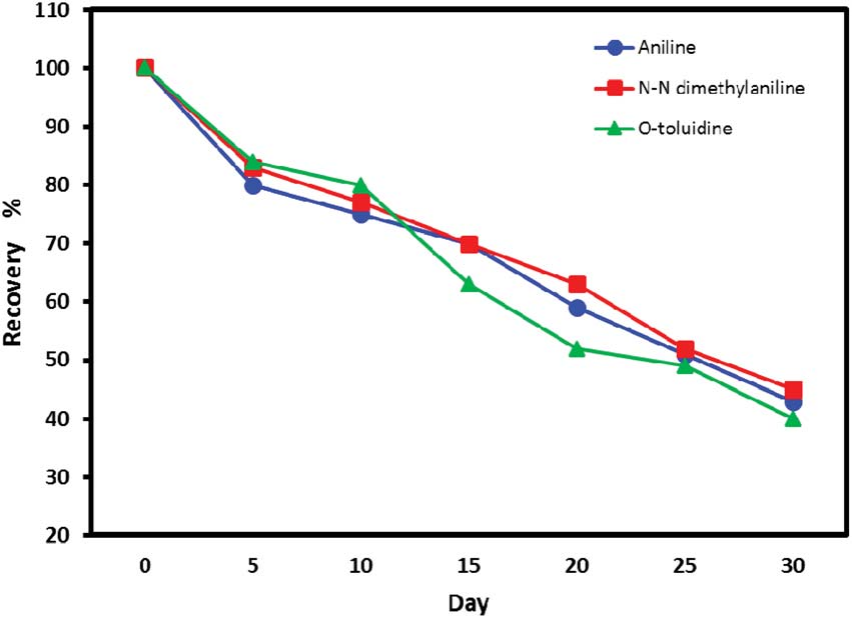
Recovery percentage of volatile aromatic amine compounds from NTD packed with UiO-66 adsorbent in the period of 1–30 days.

The results of analyte detection after 30 days were compared with the NTDs analyzed immediately. The reduced amount of the trapped analytes on the adsorbent surface was measured over time. These results are shown in Fig. 7. As results show, at room temperature, seven days after sampling, the recovery percentage of analytes for the proposed NTD was higher than 80%, but after 30 days, the concentration of the analyte reached below 50%. While NTD kept at refrigerator temperature (4 °C), no significant change in analyte concentration occurred. Which showed the suitable capability and absorption affinity of the packed adsorbent for storing the analytes. The main reason is attributed to the NTD structure that does not provide conditions for analytic escape. Because there is a very little free space inside the packed NTD, and therefore after sampling, sealing both ends of the NTD prevents the flow of air and escape of the trapped analytes. Therefore, the probability of analyte loss is less than other sampling techniques.

### Repeatability and reproduction capability

In this study, the RSD was used to determine the repeatability and reproducibility of the proposed NTD. In this part, repeatability of the proposed NTD for the sampling and analysis of the analytes of interest were evaluated at five concentrations of 0.1, 1.0, 10.0, 30.0 and 70.0 ng mL^−1^. Table 4 shows the results of repeatability and reproducibility. The RSD percentages were in the range 1.3–6.8%, which indicated suitable repeatability and accuracy for the proposed method. The repeatability of the NIOSH method (2002) for the determination of aromatic amines was reported to be 11.2–16%, which is comparable to the results of this study.

**Table 4:** Relative standard deviation percentages for evaluation of repeatability and reproducibility of NTD packed with UiO-66 adsorbent for sampling and analysis of aromatic amine compounds at different concentrations

Analytes	RSD% for a NTD at different concentrations (ng mL^−1^)					RSD% for different NTDs at a constant concentration (1.0 ng mL^−1^)		
	0.1	1	10	30	70	NTD1	NTD2	NTD3
Aniline	5.6	3.1	2.3	3.3	2.1	8.6	7.1	6.9
*N*,*N*-Dimethylaniline	1.5	2.3	3.4	4.4	3.9	7.4	8.9	9.7
*o*-Toluidine	1.3	3.3	4.8	6.8	4.8	8.2	9.9	7.6

In the present study, in order to evaluate the reproductive capability of the proposed NTD, three different NTDs were used for sampling the same concentration (1.0 ng mL^−1^) under optimal sampling and desorption conditions. As shown in Table 4, there was no significant difference between measurements, and the reproducibility of the samplers was in the acceptable range. These results demonstrated that the NTD packed with UiO-66 sorbent offered a good reproducibility and confirmed the advantage of the technique for applying as a repeatable and reproducible method. It should be noted that in the evaluation of the repeatability and reproducibility of the NTD, the experiments were repeated three times, and the standard deviations of the mean were reported.

### Method validation

In this step, the LOD and LOQ were determined experimentally by decreasing the concentrations of the analytes inside the standard chamber to reach the concentrations corresponding the signal to noise ratios of 3 and 10, respectively. The LOD values of the NTD packed with UiO-66 adsorbent for aniline, *N*,*N*-dimethylaniline and *o*-toluidine, with sampling volume 300.0 mL, were 0.02, 0.02, and 0.01 ng mL^−1^, respectively. Moreover, the LOQ values for mentioned analytes was determined to be 0.03, 0.05, and 0.05 ng mL^−1^, respectively. The results of LOD and LOQ are presented in Table 5. These results indicated that UIO-66 adsorbed packed NTD has higher sensitivity over the standard NIOSH method (NIOSH-2002).

**Table 5:** LOD, LOQ and LDR of NTD packed with UiO-66 adsorbent for the sampling and analysis of aromatic amine compounds (sampling volume 300.0 mL)

Analytes	LOD (ng mL^−1^)	LOQ (ng mL^−1^)	LDR (ng mL^−1^)	*R* ^2^
Aniline	0.02	0.03	0.01–100	0.99
*N*,*N*-Dimethylaniline	0.02	0.05	0.01–100	0.97
*o*-Toluidine	0.01	0.05	0.01–100	0.98

In a study, Amberlite XAD-2 resin has been previously used in NTD for extraction of aromatic amines. In the mentioned study, the LOD values for aniline, *N*,*N*-dimethylaniline and o-tolidine were 0.01, 0.02 and 0.01 ng mL^−1^, respectively.^3^ In another study, the LOD values for aniline and tolidine were estimated to be 6 and 70 ng L^−1^, respectively. Moreover, in a similar study, the LOQ values for aniline, *N*,*N*-dimethylaniline and o-tolidine were estimated to be 1, 1 and 10 ng L^−1^, respectively.^40^

In this study, the LDR was also evaluated as another analytical parameter. The NTD packed with UiO-66 adsorbent provided a broad LDR up to 150.0 ng mL^−1^, which demonstrated a high adsorption capacity and can be used for higher concentrations.

**Fig. 8:**
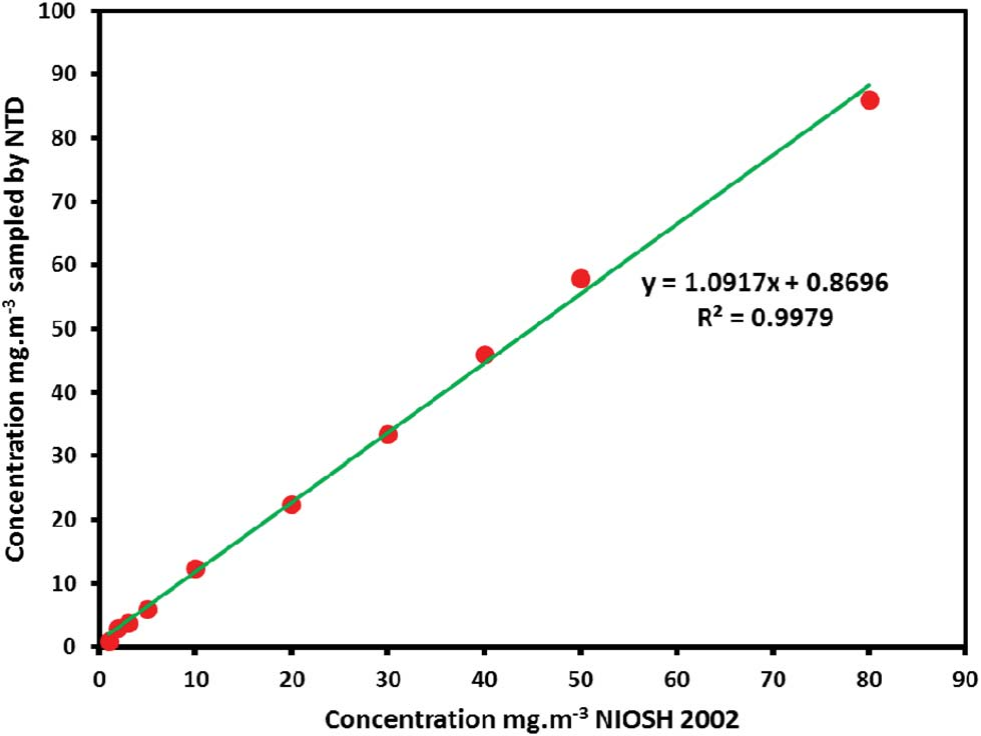
Comparison of the obtained results of the real sampling by the NTD packed UiO-66 absorbent with the reported results by standard procedure (NIOSH 2002).

The accuracy of the obtained results of the real sampling by the NTD packed UiO-66 absorbent compared with the reported results by the standard method (NIOSH 2002). Comparison of the results and the achieved *R*^2^ (0.98–0.99) showed a sufficient correlation of two methods (Fig. 8).

### Real sampling analysis

After optimization of the laboratory circumstance and determination of effective parameters on the sampling and analyzing of aromatic amines by the NTD packed UiO-66 absorbent, the efficiency of this method evaluated on the real sampling of the plastic industry containing aniline and *N*,*N*-dimethylaniline samples. The achieved results through NTD packed UiO-66 absorbent compared with the standard procedure as following in Table 6: (NIOSH 2002).

**Table 6:** Comparative results of the NTD packed UiO-66 and NIOSH 2002 methods

Analyte	NTD-UiO-66		NIOSH 2002	
	Concentration (ppm)	RSD (%)	Concentration (ppm)	RSD (%)
Aniline	3.9	11.4	2.1	12.1
*N*,*N*-Dimethylaniline	6.3	10.1	5.2	11.2

## Conclusion

In this study, NTD packed with UiO-66 adsorbent, which has high absorption capacity and selectivity, was developed. This study was conducted both in the laboratory and in the field. The number of experiments was determined using the RSM by CCD, and the sampling parameters (temperature and relative humidity) and desorption parameters (desorption time and temperature) were optimized for the sampling and analysis of aromatic amines. The results demonstrated that the highest peak responses were observed at desorption time of 3 minutes and the desorption temperature of 270 °C. In this study, the NTD packed with UiO-66 adsorbent offered suitable repeatability (RSD: 1.3–6.8%) and acceptable reproducibility (RSD: 1.3–9.7%). The proposed NTD offered a very high sensitivity for determination of aromatic amines, which the values of LODs and LOQs for the mentioned analytes were in the range 0.01–0.02 and 0.03–0.05 ng mL^−1^, respectively. The recovery percentage of NTD packed with UiO-66 adsorbent was higher than 90% after 30 days at 4 °C for the studied analytes. Therefore, this type of NTD can be used for the sampling and analysis of aromatic amines at trace levels in workplaces with high accuracy and sensitivity.

## Conflicts of interest

No potential conflict of interest was reported by the authors.

## Supplementary Material
